# Extreme precipitation patterns in the Asia–Pacific region and its correlation with El Niño-Southern Oscillation (ENSO)

**DOI:** 10.1038/s41598-023-38317-0

**Published:** 2023-07-08

**Authors:** Dong An, Jakob Eggeling, Linus Zhang, Hao He, Amir Sapkota, Yu-Chun Wang, Chuansi Gao

**Affiliations:** 1 Division of Water Resources Engineering, Faculty of Engineering (LTH), Lund University, Lund, Sweden; 2 Aerosol and Climate Laboratory, Division of Ergonomics and Aerosol Technology, Department of Design Sciences, Faculty of Engineering (LTH), Lund University, Lund, Sweden; 3 Department of Atmospheric and Oceanic Science, University of Maryland, College Park, MD 20742 USA; 4 Department of Epidemiology and Biostatistics, School of Public Health, University of Maryland, College Park, MD 20742 USA; 5 Department of Environmental Engineering, College of Engineering, Chung Yuan Christian University, 200 Chung-Pei Road, Zhongli, 320 Taiwan

**Keywords:** Hydrology, Climate-change impacts

## Abstract

In the Asia–Pacific region (APR), extreme precipitation is one of the most critical climate stressors, affecting 60% of the population and adding pressure to governance, economic, environmental, and public health challenges. In this study, we analyzed extreme precipitation spatiotemporal trends in APR using 11 different indices and revealed the dominant factors governing precipitation amount by attributing its variability to precipitation frequency and intensity. We further investigated how these extreme precipitation indices are influenced by El Niño-Southern Oscillation (ENSO) at a seasonal scale. The analysis covered 465 ERA5 (the fifth-generation atmospheric reanalysis of the European Center for Medium-Range Weather Forecasts) study locations over eight countries and regions during 1990–2019. Results revealed a general decrease indicated by the extreme precipitation indices (e.g., the annual total amount of wet-day precipitation, average intensity of wet-day precipitation), particularly in central-eastern China, Bangladesh, eastern India, Peninsular Malaysia and Indonesia. We observed that the seasonal variability of the amount of wet-day precipitation in most locations in China and India are dominated by precipitation intensity in June–August (JJA), and by precipitation frequency in December–February (DJF). Locations in Malaysia and Indonesia are mostly dominated by precipitation intensity in March–May (MAM) and DJF. During ENSO positive phase, significant negative anomalies in seasonal precipitation indices (amount of wet-day precipitation, number of wet days and intensity of wet-day precipitation) were observed in Indonesia, while opposite results were observed for ENSO negative phase. These findings revealing patterns and drivers for extreme precipitation in APR may inform climate change adaptation and disaster risk reduction strategies in the study region.

## Introduction

Ongoing climate change is impacting regional precipitation pattern across the globe^1^. Higher air temperature and evaporation rate lead to greater accumulation of water vapor in the air, resulting in increased storms, more intense precipitation as well as land surface drying due to prolonged drying periods^2^. With drier land surfaces and increased precipitation, the land becomes prone to both droughts and floods^2–4^. Large scale climate phenomenon can influence precipitation patterns across the globe and influence burden of food and waterborne diseases, particularly in low- and middle-income countries (LMICs)^5–8^.

The meteorological factors influencing precipitation patterns in the Asia–Pacific region (APR) are exceedingly intricate, characterized by interdependencies among them. Monsoon plays a crucial role that can impact both spatial and temporal variations of precipitation, as described previously^9^. Initially, the monsoon begins with rainfall surges in the South China Sea in May which extends to the Arabian Sea and the Bay of Bengal. This rainband initiates the continental Indian rainy season, the Chinese Mei-yu and the Japanese Baiu in June and matures over July and August, making June–August important months for studying the large-scale precipitation^9^. In addition, El Niño-Southern Oscillation (ENSO) holds significant influence on weather patterns, temperature anomalies, precipitation regimes, and atmospheric circulation patterns^10–13^. ENSO’s impacts can be complicated by other large scale weather phenomenon such as the Indian Oceanic Dipole (IOD)^14^, North Atlantic Oscillation (NAO)^15^ and the Pacific Decadal Oscillation (PDO)^16^. Previous research has raised concern regarding a weakened correlation between ENSO and the Indian summer monsoon^17^. A more recent study indicated that each unit change in IOD currently has a proportionately greater impact on Indian monsoon than ENSO^18^. Previous studies investigating the influence of ENSO on precipitation variations in APR have elucidated the spatiotemporal patterns across diverse areas. For instance, Tamaddun et al.^19^ suggested that the ENSO positive phases have greater influence on northern India’s monsoon precipitation pattern compared to ENSO negative or neutral phase. Sigdel and Ikeda^20^ concluded that summer monsoon rainfall over Nepal shows significant correlation with ENSO and early (late) onset timing of monsoon can induce more (less) precipitation in June. Wahiduzzaman and Luo^21^ explored the seasonal variability of rainfall anomalies during two types of ENSO events in Bangladesh. They observed negative rainfall anomaly in western of Bangladesh during CP (central Pacific) El Niño events, while positive rainfall anomaly was seen in most parts of Bangladesh during EP El Niño (equatorial eastern Pacific), while La Niña showed positive rainfall anomalies in northern Bangladesh. Likewise, Li et al.^22^ noted that extreme precipitation events in China is more common in El Niño phases during DJF and MAM, and in La Niña phases during JJA and SON. Zhang et al.^23^ noted that southern China experiences an increase in precipitation amount in DJF, MAM and SON during El Niño mature phase, while both northern and southern China experience decrease in precipitation during JJA. Similarly, western and central mountain area of the Taiwan region experience low (high) precipitation extremes during El Niño (La Niña), and it is the opposite for northern and eastern area^24^. The correlation between ENSO and precipitation events in Vietnam show a latitudinal dependency, which is stronger in the south^25^. La Niña appears to bring enhanced rainfall during DJF and El Niño tends to be associated with reduced rainfall in JJA^25,26^. In Malaysia, the impact of ENSO on precipitation is dependent on the intensity of the ENSO event itself, with strong (moderate) La Niña related to significant decrease (increase) in wet precipitation extremes over the Peninsular Malaysia during DJF^27^. Supari et al.^28^ concluded that there is a tendency towards a wetter condition covering the northern part of Indonesia, and a drying trend at country level (mostly contributed by a southern region of the country), characterized by a significant increase in the consecutive dry days (*CDD*) during JJA, SON and MAM. Supari et al.^29^ further suggested that during the El Niño developmental period in JJA and SON, dry conditions are experienced throughout entire Indonesia. However, from SON a wet anomaly appears over northern Sumatra, later expanding eastward during DJF and MAM.

Most of the above studies have investigated the impacts of ENSO on variations of precipitation by establishing relations between historical precipitation records and different ENSO phases at national or regional scale over APR. Based on existing research findings, the principal objective of this study is to identify the spatial–temporal patterns of various intra-annual extreme precipitation characteristics (amount of precipitation, number of wet days, and intensity of precipitation) and their relationships with different ENSO phases in order to comprehensively enhance the understanding of their associations over the study region. The potential regional consistency and significant anomalies may offer implications for improving water resources management and provide an opportunity to develop public health early warning systems with sub-seasonal to seasonal lead times. In this study, trend analysis (1990–2019) is conducted using 11 extreme precipitation indices introduced by the Expert Team on Climate Change Detection and Indices (ETCCDI)^30^ followed by statistical analysis to detect the dominant factors governing precipitation amount. In addition, composite analysis is applied to identify the influence of ENSO on the precipitation characteristics in APR.

## Results

### Spatial distribution of extreme precipitation trends

**Figure 1:**
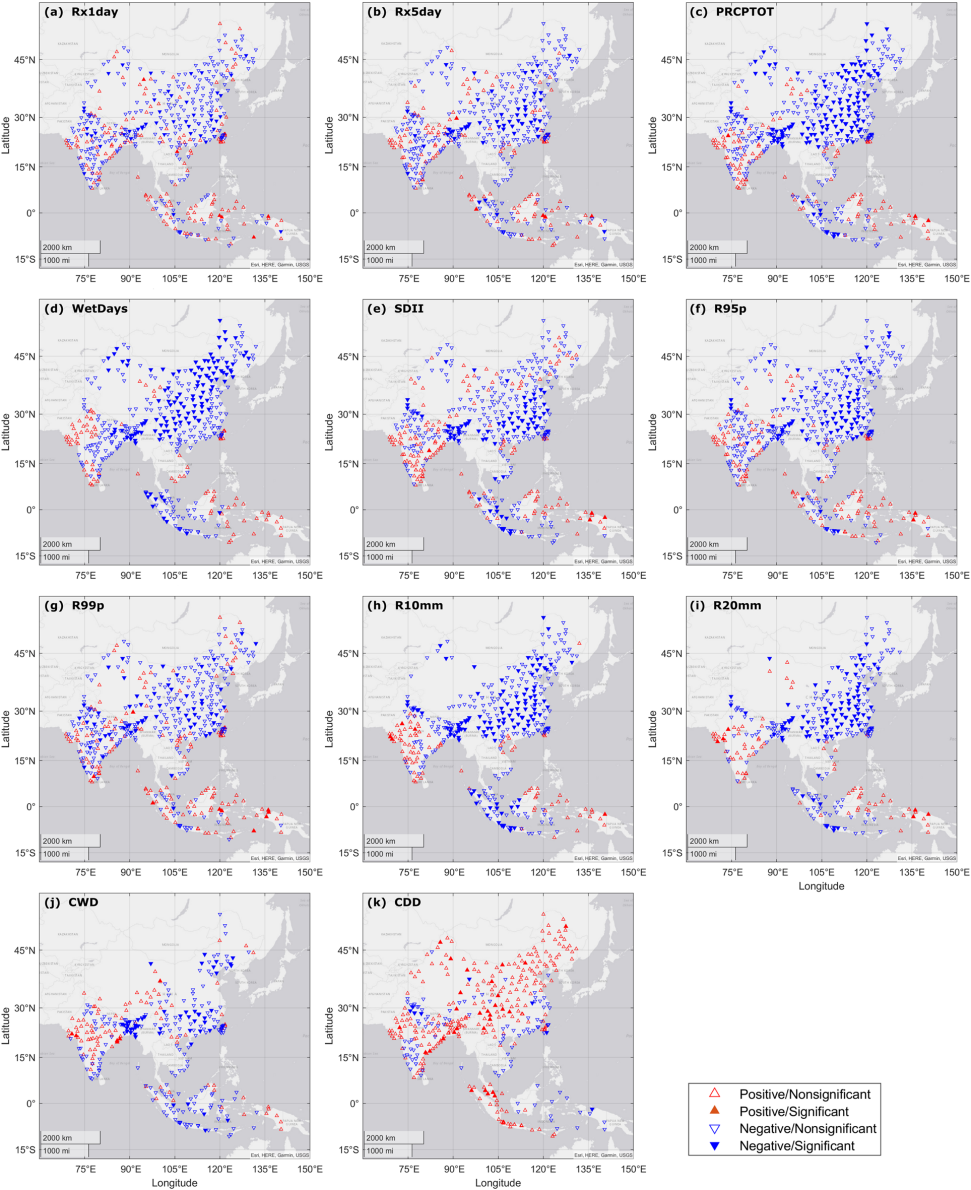
Spatial distribution of the annual trends (1990–2019) of the 11 indices: (**a**) *Rx1day*, (**b**) *Rx5day*, (**c**) *PRCPTOT*, (**d**) *WetDays*, (**e**) *SDII*, (**f**) *R95p*, (**g**) *R99p*, (**h**) *R10mm*, (**i**) *R20mm*, (**j**) *CWD* and (**k**) *CDD* based on the iterative Mann–Kendall trend test. Upward (downward) triangles indicate positive (negative) trends, solid triangles indicate the trends at 95% significance level. Locations with no trend are not shown.

We computed eleven extreme precipitation indices at an annual scale for all 465 locations (Table 1 in “Methods” section, Supplementary Fig. 1). Majority of indices revealed decreasing trend during the study period including *PRCPTOT*, *WetDays*, *R95p*, *R10mm* and *R20mm*. *CDD* showed the most pronounced increasing trend while *PRCPTOT* had the most decreasing trend, at both 0.05 and 0.10 significance levels. The spatial patterns of long-term trends (1990–2019) for the 11 indices in APR are shown in Fig. 1.

**Table 1:** The extreme precipitation indices used in this study.

Index	Definitions	Units	Category
PRCPTOT	Total precipitation from wet days during a specific period (Annual/seasonal)	mm	i
SDII	Average precipitation intensity from wet days	mm/day	i
WetDays	Count of days with daily precipitation depth ≥ 1 mm during a specific period	days	i
Rx1day	Maximum 1-day precipitation during a specific period	mm	i
Rx5day	Maximum 5-day precipitation during a specific period	mm	i
R95p	Total annual precipitation from days with daily precipitation > 95th percentile	mm	ii
R99p	Total annual precipitation from days with daily precipitation > 99th percentile	mm	ii
R10mm	Annual count of days when precipitation ≥ 10 mm	days	ii
R20mm	Annual count of days when precipitation ≥ 20 mm	days	ii
CWD	Maximum length of wet spell: maximum number of consecutive days with RR ≥ 1 mm	days	iii
CDD	Maximum length of dry spell: maximum number of consecutive days with RR < 1 mm	days	iii

For the indicators of general precipitation pattern, significant decreasing trends in *Rx1day* and *Rx5day* (Fig. 1a,b) were observed in central China, Bangladesh, and eastern India (around 30°N). Locations with increasing trends were observed mainly in western China, southern part of Taiwan region, coastal area of India and eastern part of Malaysia and Indonesia. To detect seasonal extreme precipitation trends over the study period, we performed additional trend analysis at a seasonal scale for *Rx1day* and *Rx5day*, which showed similar patterns (Supplementary Figs. 2, 3). For most locations in China, India, Bangladesh and Vietnam, the similarity in patterns between annual scale and JJA may be due to the occurrence of maximum 1-day/5-day precipitation in this season (JJA). We observed increasing trends in north-east and north-west of China and southern India in MAM and DJF. During the study period, *PRCPTOT* and *WetDays* had similar spatial patterns that differed from *SDII* (Fig. 1c–e). We observed increasing trends in *PRCPTOT* and *WetDays* in north-west China, western and southern India, southern part of Taiwan region, eastern Malaysia, and Indonesia, while, decreasing trends were observed in north-east to south-west China, eastern India, Bangladesh, and eastern Indonesia. Compared to *PRCPTOT* and *WetDays*, spatial pattern of *SDII* showed less significant decreasing trends in central and northern China.

Significant decreasing trends in *R95p* and *R99p* were observed in central China, northern and eastern India, and Bangladesh (Fig. 1f,g), while increasing trends were observed in western and southern India, southern Taiwan, eastern Malaysia, Indonesia, and northern China. Number of days with extreme precipitation (*R10mm* and *R20mm*) decreased in north-eastern and central China, northern and eastern India, Bangladesh, and eastern Malaysia and Indonesia (Fig. 1h,i). Note that for some locations, daily precipitation did not reach 99% percentile threshold in specific years, resulting in a trend calculated as 0, as per the methodology of Sen’s slope calculation (see “Methods” section). Consequently, there were less trends detected in *R99p* compared to *R95p*. Similarly, for some locations in arid areas, insufficient *R20mm* values precluded reliable calculation of trend.

We observed significant decreasing trends for *CWD* at 50 locations (Fig. 1j, Supplementary Table 1), distributed across north-eastern and southern China, Bangladesh, eastern India, and southern Indonesia. Increasing *CWD* trends were observed mainly in western China, central and eastern India, and eastern Indonesia. Most locations displayed increasing trends in *CDD* (Fig. 1k), which indicates a prolonged consecutive dry condition in most parts of study area. We observed 29 significant increasing trends in *CDD* across south-western China, eastern India and Peninsular Malaysia, while decreasing trends were observed in south-eastern coast of China, northern and southern India, southern Vietnam and eastern Indonesia.

### Dominance analysis of precipitation variability at annual and seasonal scale

We conducted dominance analysis at annual scale (Supplementary Fig. 4), and observed significant (p ≤ 0.05 for F-test) regression coefficients for all locations. Approximately 85% of locations were above the 45° black line (S_i > S_n values; Supplementary Fig. 4a), indicating the dominance of precipitation intensity for most of the locations in study area. Only 15% of locations had a larger S_n value, indicating dominance of precipitation frequency. Spatial distribution of dominance analysis results illustrates a wide spread of locations dominated by precipitation intensity (Supplementary Fig. 4b). The 77 locations dominated by precipitation frequency are primarily located in western and northern China and southern Indonesia. Besides, several locations in north-west India and south-east China are also identified as frequency dominated.

**Figure 2:**
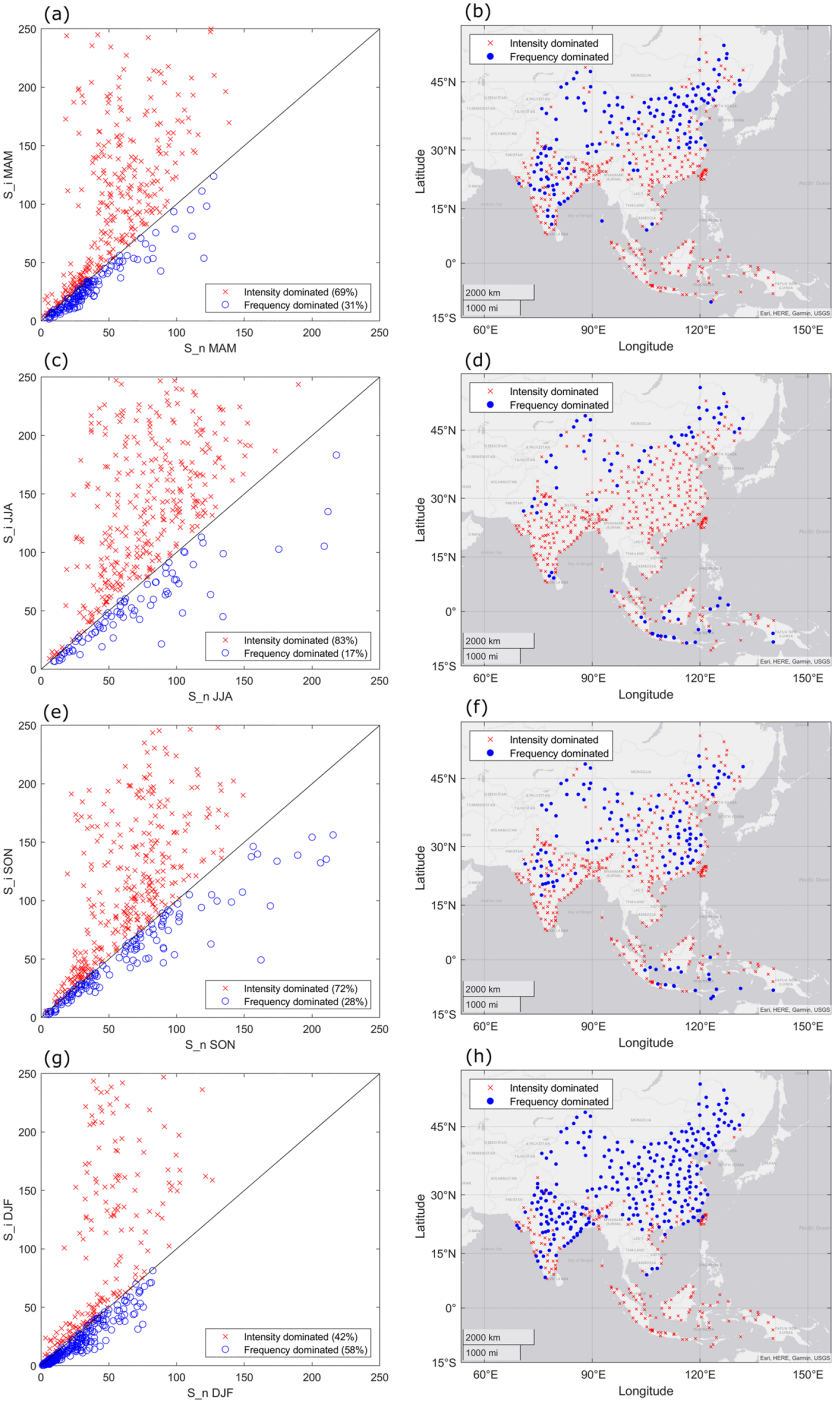
The (**a**) scatter plot of seasonal S_n and S_i and (**b**) spatial distribution of locations with dominances of intensity and frequency. The 45° line represents the condition S_n = S_i. Locations marked as red cross indicates the dominance of intensity (S_i > S_n), the ones marked as blue dots indicated the dominance of frequency (S_n > S_i).

We performed similar analyses at a seasonal scale for MAM, JJA, SON and DJF (Fig. 2). As depicted in scatter plots (Fig. 2a,c,e,g), seasonal precipitation amounts for most locations are dominated by precipitation intensity in MAM (69%), JJA (83%) and SON (72%). In JJA, only 17% of locations are found to be dominated by frequency. However, unlike the other three seasons, the variability of precipitation amount is dominated by precipitation frequency for most locations in DJF (58%). For MAM and SON, the ratios of domination factors are similar. Spatial patterns of the dominance distribution are displayed in Fig. 2b,d,f,h. In China, the north–south spatial pattern dominated by different factors was found during MAM and JJA. Most locations are dominated by frequency in DJF. Locations in central India were dominated by frequency in MAM and SON. Most locations are found to be dominated by intensity in JJA, but by frequency in DJF. For Bangladesh, Nepal and eastern India, all locations are dominated by intensity in MAM, JJA and SON. In Vietnam, all locations are dominated by intensity in JJA and SON. Locations in Malaysia and Taiwan region are dominated by intensity at all seasons. In Indonesia, locations in central and the south are dominated by frequency in JJA and SON, and all locations are dominated by intensity in MAM and DJF. All the coefficients of regression for all seasons are statistically significant (p ≤ 0.05 for F-test) for all locations.

### Influence of ENSO on seasonal precipitation variability

**Figure 3:**
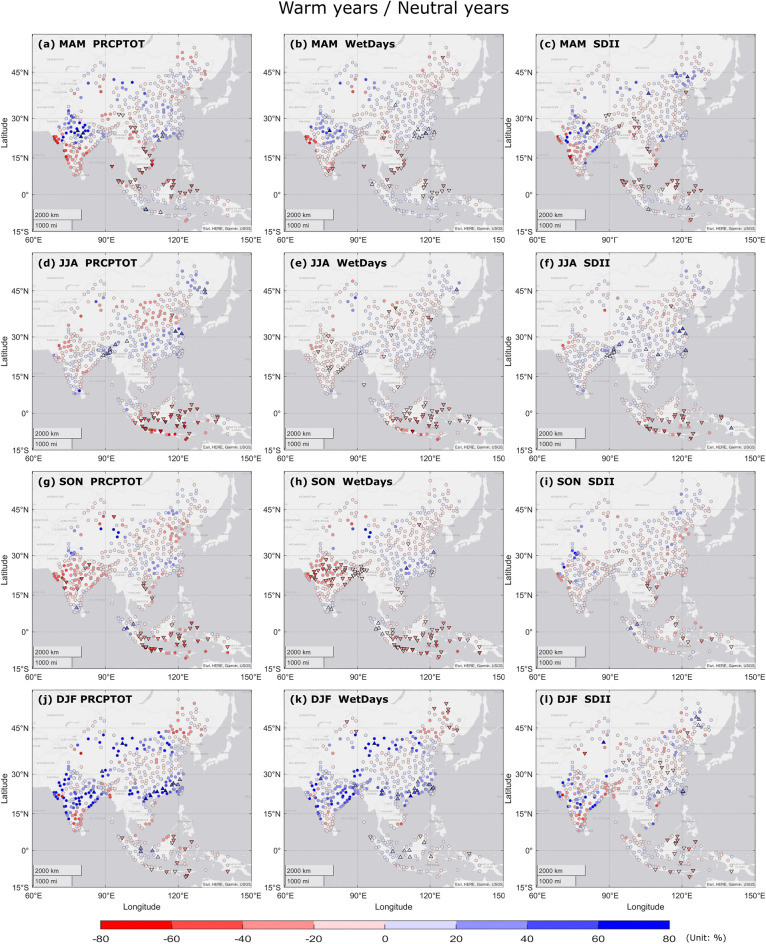
Spatial patterns of seasonal *PRCPTOT*/*WetDays*/*SDII* differences between warm years/neutral years (El Niño phase/normal phase) during 1990–2019, in (**a–c**) MAM, (**d–f**) JJA, (**g–i**) SON, (**j–l**) DJF. Blue (red) color denotes for positive (negative) seasonal anomaly. Triangles indicate significant differences at 95% significance level between warm and neutral episodes of ENSO. Circles indicate non-significant differences.

The relative differences between El Niño phase and ENSO neutral phase in *PRCPTOT*, *WetDays* and *SDII* are displayed in Fig. 3. During El Niño MAM (Fig. 3a–c), locations in Vietnam, south-western China, eastern Malaysia and northern Indonesia showed significant decrease in all precipitation indices than neutral phase. North-central India experienced an increase in all indices during El Niño episodes while the south-western coast showed a strong decrease (not significant). Significant positive anomalies of precipitation amount and wet days were observed in southern part of China. During El Niño JJA (Fig. 3d–f), northern China received less amount of precipitation and wet days (with less significant differences). Most locations in central and eastern Indonesia showed significant negative anomalies in all three indices, while western Indonesia experienced fewer wet days and weaker precipitation intensity. Negative anomalies were observed at the locations in north-western India for all precipitation indices, and significant positive anomalies in precipitation amount and intensity were identified in north-eastern India and Bangladesh. During El Niño SON (Fig. 3g–i), most areas in India, Bangladesh, Vietnam, Taiwan region and central and eastern Indonesia showed less precipitation and wet days. Positive anomalies of precipitation amount and intensity were detected in northern India. During El Niño DJF (Fig. 3j–l), locations in southern and northern China, central and northern India, and northern Vietnam experienced more precipitation and wet days than ENSO neutral phase.

**Figure 4:**
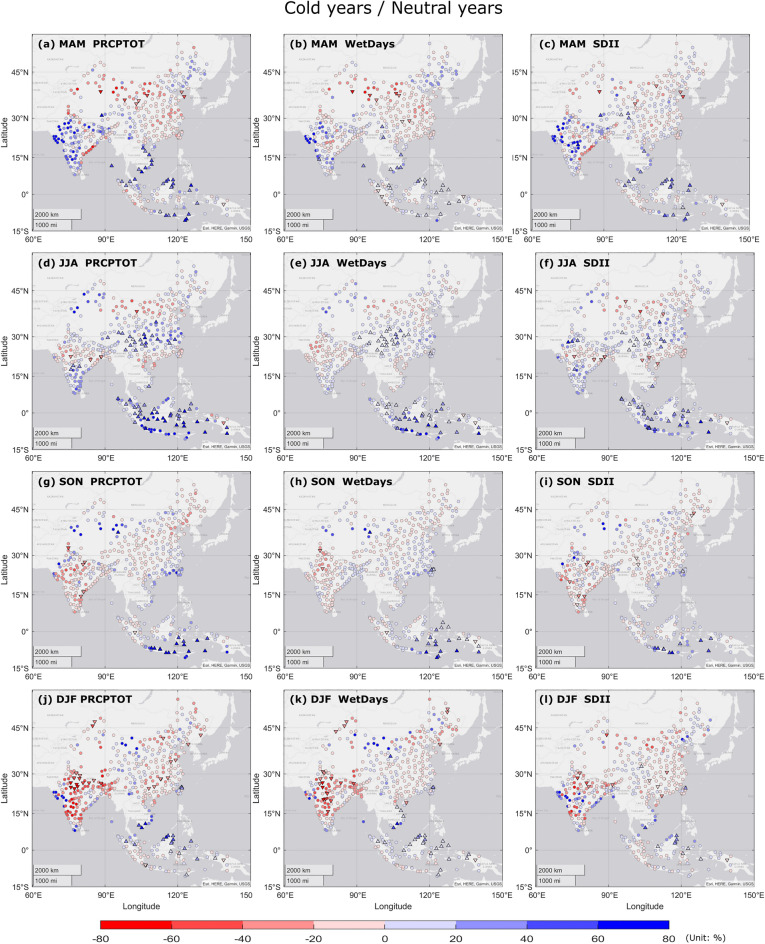
Spatial patterns of seasonal *PRCPTOT*/*WetDays*/*SDII* differences between cold years/neutral years (La Niña phase/normal phase) during 1990–2019, in (**a–c**) MAM, (**d–f**) JJA, (**g–i**) SON, (**j–l**) DJF. Blue (red) color denotes for positive (negative) seasonal anomaly. Triangles indicate significant differences at 95% significance level between cold and neutral episodes of ENSO. Circles indicate non-significant differences.

The relative differences between La Niña phase and ENSO neutral phase in *PRCPTOT*, *WetDays* and *SDII* are displayed in Fig. 4. During La Niña MAM (Fig. 4a–c), positive anomalies of all three indices were found in Vietnam, north-western and south-eastern India, eastern Malaysia and southern Indonesia. Negative anomalies were observed in northern China and south-eastern coast of India. In JJA during La Niña (Fig. 4d–f), significant positive anomalies were observed in nationwide Indonesia and south-western China. In northern China, northern Vietnam and central India, negative anomalies of precipitation indices were found. During La Niña SON (Fig. 4g–i), significant positive anomalies were observed in southern and eastern Indonesia, positive anomalies were also discovered in north-western China and Taiwan region. Negative anomalies of precipitation amount and wet days were observed in central and southern India. In DJF (Fig. 4j–l), most of locations in India experienced less precipitation amount and wet days during La Niña phase than neutral phase.

## Discussion

This study revealed distinct spatial and temporal trends of multiple extreme precipitation indices in APR. Trend analysis results are often influenced by the study period. Our study period is confined to the post climate regime shift of 1970s^31–33^. Significant change has been observed for precipitation in East Asia since the climate shift^34^. Gong et al.^35^ reported that post climate regime shift is characterized by excessive rainfall in JJA over the Yangtze River valley but dry anomalies over the northern China. Before the climate shift, however, the pattern was comparable but rainfall anomalies switch signs. Our study showed that the trends of *Rx1day* and *Rx5day* increased in the Yangtze River valley but decreased over northern China in JJA and SON. The increasing trends of extreme precipitation indices observed in northwestern China is consistent with the findings of Lu et al.^36^. Increasing trends were also found in southern India for the indices of general precipitation pattern, e.g., *PRCPTOT*, *SDII*, *WetDays*, in agreement with the findings of Dash et al.^37^. Similar spatial patterns of trends in R95p and *CDD* were also reported in Indonesia by Supari et al.^28^.

The findings from dominance analysis indicate that for most locations in the study area, the precipitation amount is mainly affected by the strength of precipitation intensity, rather than the number of precipitation days at annual scale. The precipitation amounts in Bangladesh, Taiwan region, northern Vietnam and Indonesia (western, northern and far eastern in Papua) are dominated by precipitation intensity for all seasons. It should be noted that the results of the dominance analysis are sensitive to the selected threshold which defines a rainy day. In our analysis, we used 1 mm/day to balance both humid and arid areas. However, such stringent threshold may be insufficient to define a rainy day in humid regions. By increasing the threshold, the number of locations with dominating intensity will decrease, and the number of locations with dominating frequency will increase. With the threshold 1 mm/day, our findings for spatial pattern of precipitation for JJA (China) is similar to the one reported by Lu et al.^38^.

Positive (negative) phase of ENSO events has been linked to reduced (excess) Indian summer monsoon (June, July, August and September) rainfall^39^, even though their relationship is in the weakening stage during the recent decades^17^. Our results indicate that almost whole India experiences less precipitation days in JJA during El Niño; the south of India shows positive anomalies in precipitation amount and number of precipitation days during La Niña phase. Previous research has indicated increased (decreased) wintertime (December, January, February and March) precipitation over northwestern India during warm (cold) phase of ENSO^40^. Our results agree with this, it is worth noting that during El Niño phase there is a widespread drying condition in the season of SON. Our findings suggest that the precipitation in Nepal during MAM, JJA and SON follows the pattern that less (more) precipitation amount is experienced during El Niño (La Niña), which is coherent with results of Sigdel et al.^20^. We identify spatial patterns in Bangladesh that is consistent with the results of Wahiduzzaman and Luo^21^ associated with EP (equatorial eastern Pacific) ENSO. Negative anomaly of precipitation amount is found in south-eastern of Bangladesh during DJF, positive anomaly is evident nationwide during JJA. The study of Ehsan et al.^41^ also confirmed that the relationship between Bangladesh summer monsoon (June–September) rainfall and ENSO is asymmetric: a nationwide weak positive correlation was found between summer monsoon rainfall and ENSO warm phase, however, the relationship was more varied during the ENSO cold phase. Our findings suggest that El Niño strongly intensifies the amount of precipitation and number of precipitation days during DJF in the south and south-eastern part of China, consistent with the findings of Gao et al.^42^ and Zhang et al.^23^. However, under the influence of El Niño, both DJF precipitation and the number of precipitation days in central and north-eastern China are weakened, which is slightly different from the conclusion of Gao et al.^42^. Besides, our results show positive anomalies in north-eastern China for all the precipitation indices in JJA during El Niño, which supports the finding of Han et al.^43^ about the strengthened relationship between ENSO and summer precipitation over north-eastern China after late 1990s. In Taiwan Region, both precipitation amount and intensity tend to increase during El Niño and decrease during La Niña in MAM, while decrease during El Niño and increase during La Niña in SON. Additionally, a general increasing trend in precipitation amount and intensity in DJF is observed during both El Niño and La Niña episodes. We observed a general latitudinal dependency where southern Vietnam becomes drier (wetter) during El Niño (La Niña) for all seasons. This is consistent with findings reported by Vu-Thanh et al.^25^ and Nguyen et al.^26^. Juneng et al.^44^ and Supari et al.^28^ concluded that El Niño (La Niña) enhances dry (wet) conditions in Malaysia. This pattern was observed in our study especially during MAM, JJA and DJF. Meanwhile, in southern Malaysia it shows anomalies opposite to the general pattern that El Niño (La Niña) brings wetter (dryer) condition. Supari et al.^29^ identified a west–east divide in Indonesia where El Niño (La Niña) increased wet (dry) conditions in the west and dry (wet) in the east. Generally, similar patterns are observed in our results in all seasons except for JJA, when the wet conditions are enhanced by La Niña all over Indonesia. Besides, opposite anomalies between the north and south of Sumatra for all extreme precipitation indices are detected for MAM and SON during El Niño and La Niña phases.

This work utilized ERA5 reanalysis data for analyzing extreme precipitation characteristics and how they are related to phases of ENSO. ERA5 is produced using an advanced assimilation system and parameterization scheme at high spatiotemporal resolution. While the use of ERA5 data for extreme precipitation analysis poses challenges, e.g., Jiang et al.^45^ pointed out that ERA5 tends to underestimate moderate and heavy precipitation events over Chinese Mainland; Kim et al.^46^ noted that the contribution of model resolution remains debatable regarding extreme precipitation over Asia monsoon region. For these reasons, it is essential to calibrate reanalysis data with ground observations for the improvement of its accuracy due to the complexity of precipitation phenomenon when station data are complete and available. Despite these limitations, our results suggest that phases of ENSO are related to seasonal extreme precipitation events in APR. Since ENSO can be predicted reasonably well with a seasonal lead time, it may provide useful information for public health early warning system for diseases that are influenced by extreme precipitation.

## Methods

We downloaded whether station data from Oceanic and Atmospheric Administration (NOAA) Global Historical Climatology Network (GHCN: https://www.ncei.noaa.gov/products/land-based-station/global-historical-climatology-network-daily). Overall, there were 465 weather stations during 1990–2019 in our study area over eastern and southern Asia (Supplementary Fig. 1). However, we encountered significant missing data, particularly during the earlier period. For this reason, we used fifth-generation atmospheric reanalysis of the European Center for Medium-Range Weather Forecasts (ERA5) reanalysis product for our main analysis and included the station level data as supplementary material. The 465 locations included in our analysis represent ERA5 grids covering the location of the 465 GHCN stations. In this study, the annual scale analysis is performed following the definition of a calendar year (January 1st to December 31st). The seasons are defined as: MAM (March, April, May), JJA (June, July, August), SON (September, October, November), and DJF (December, January, February).

### Trend analysis

We computed a total of 11 extreme precipitation indices (Table 1) following ETCCDI recommendation. The 11 indices can be grouped into three different categories: (i) indices of general precipitation pattern: *PRCPTOT*, *SDII*, *Rx1day*, *Rx5day*, *WetDays*; (ii) indices of extreme precipitation: *R95p*, *R99p*, *R10mm*, *R20mm*; and (iii) others: *CWD*, *CDD*.

To examine the significance of trends in the extreme precipitation indices, we used the non-parametric Mann–Kendall’s (MK) trend test^47,48^. Original MK trend test tends to reject the null hypothesis of no trend more often than specified by the significance level when the data have a positive autocorrelation^49^. To address this concern, we followed a method previously described by Yue et al.^50^ that can eliminate the influence of the trend on the serial correlation by removal of a trend component from a time series prior to pre-whitening. We then applied Theil-Sen approach^51,52^ to evaluate the slopes of trends. We define significance at the 0.05 level. The iterative-based MK test was conducted using the R package ‘zyp’^53^.

### Dominance analysis of intensity and frequency in precipitation variability

We examined the influence of frequency and intensity of annual and inter-annual variability in precipitation amount by using a dominance analysis method described by Lu et al.^38,54,55^. The threshold to define a wet day was set as 1 mm/day, any days with daily precipitation above this threshold were included in the analysis. We calculated annual and seasonal number of wet days, precipitation amount and average precipitation intensity on wet days to establish the method.

For a certain period, number of wet days (with daily precipitation depth over 1 mm, denoted as *N*_*1mm*_), total precipitation amount on wet days (*P*_*tot*_), and average precipitation intensity of wet days (*I*_*wet*_) followed the relation as ${I}_{wet} = {P}_{tot}/{N}_{1mm}$. It should be noted that they correspond to *WetDays*, *PRCPTOT* and *SDII* at an annual scale as Table 1 shows. For ease of understanding, we retained the notation of all three indices for analysis at seasonal scale as seasonal *WetDays*, seasonal *PRCPTOT* and seasonal *SDII*. *P*_*tot*_, *N*_*1mm*_ and *I*_*wet*_ approximately followed a linear relation as Eq. (1):

1 $${P}_{tot} =a{\times N}_{1mm} +b\times {I}_{wet} +c,$$

where a and b are the changing rates for number of wet days and average precipitation intensity on wet days, which represent the precipitation frequency and intensity, respectively. The coefficients *a* and *b* can be expressed as $a = \partial {P}_{tot}/\partial {N}_{1mm}$ and $b = \partial {P}_{tot}/\partial {I}_{wet}$. With the data of *P*_*tot*_, *N*_*1mm*_ and *I*_*wet*_, the coefficients a, b and c were estimated by least square method.

As suggested by Lu et al., the products of the change rates (*a* and *b*) and the corresponding variation scales (the standard deviations determined by data series of *N*_*1mm*_ and *I*_*wet*_) can be used to measure, respectively, the scales of changes in *P*_*tot*_ induced by the variations of *N*_*1mm*_ and *I*_*wet*_. The two measures were expressed by Eqs. (2) and (3):

2 $$S\_n \equiv |\partial {P}_{tot}/\partial {N}_{1mm}| \times \sigma {N}_{1mm},$$

3 $$S\_i \equiv |\partial {P}_{tot}/\partial {I}_{wet}| \times \sigma {I}_{wet},$$

where $\sigma {N}_{1mm}$ and $\sigma {I}_{wet}$ are the standard deviations of data series of *N*_*1mm*_ and *I*_*wet*_. If $S\_n$ > $S\_i$, the variability of total precipitation amount *P*_*tot*_ is dominated by precipitation frequency *N*_*1mm*_, otherwise, by precipitation intensity *I*_*wet*_.

### Influence of ENSO on inter-annual precipitation variability

We investigated the impacts of ENSO events on seasonal precipitation variations in APR, particularly the warm and cold episodes of ENSO on seasonal precipitation variations. The warm episodes of ENSO (El Niño) and cold episodes of ENSO (La Niña) were defined as one standard deviation above/below the seasonal SST anomaly in Niño 3.4 area (5° N–5° S, 170°–120° W), respectively. The result is shown in Supplementary Table 3.

Seasonal *WetDays*, seasonal *PRCPTOT* and seasonal *SDII* computed in the previous section were used as indices for detecting the influence of ENSO on seasonal precipitation variability. For a specific index, the relative difference between seasonal precipitation indices of the positive (or negative) years to neutral years (${R}_{ij}$) was computed by Eq. (4):

4 $${R}_{ij}= \left(\frac{\overline{{P }_{Eij}}}{\overline{{P }_{Nij}}}-1\right) \times 100\mathrm{\%},$$

where $\overline{{P }_{Eij}}$ denotes the average seasonal precipitation indices of the ENSO positive (or negative) years in the *j*th season at the *i*th location, and $\overline{{P }_{Nij}}$ denotes the average seasonal precipitation indices of the neutral years in the *j*th season at the *i*th location. The neutral years refers to the years other than ENSO positive or negative years.

Wilcoxon rank-sum test^56^ was adopted to test the significance of equality in medians for seasonal precipitation indices between ENSO positive (or negative) years and neutral years at 0.05 significance level. The Wilcoxon rank-sum test, also known as the Mann–Whitney U Test, is a non-parametric statistical test used to compare the equality of population medians of two independent samples (or to check if they originate from the same distribution). The test works by ranking all the observations from both samples from smallest to largest, and then comparing the sums of the ranks from each sample. The null hypothesis for the test is that the medians of both samples are equal. If the test results in a p-value less than a pre-specified level (0.05 in this study), the null hypothesis can be rejected, suggesting the medians of two samples can be regarded as different from each other significantly.

## Supplementary Information

Supplementary Information.

## Data Availability

This study utilizes ERA5 reanalysis data (1990–2019)^57^ sampled over the national Oceanic and Atmospheric Administration (NOAA) Global Historical Climatology Network (GHCN) 465 weather stations for the study area over eastern and southern Asia (Supplementary Fig. 1). ERA5 data is available from the Copernicus website (https://www.ecmwf.int/en/forecasts/datasets/reanalysis-datasets/era5). The data covers the Earth on a 0.25° grid (~ 30 km). To quantify the state of ENSO, NOAA Niño 3.4 SST Index was used (available at: http://www.cpc.ncep.noaa.gov). It is calculated from averaged sea surface temperature over the area 5° N–5° S, 170°–120° W using HadISST1 SST dataset.

## Code availability

Code used for this study is available upon reasonable request from the corresponding author.
