# Supplementary material for: Extreme precipitation patterns in the Asia–Pacific region and its correlation with El Niño-Southern Oscillation (ENSO)
Source: Sci Rep. 2023 Jul 8;13:11068. doi: 10.1038/s41598-023-38317-0 (PMC10329631; doi:10.1038/s41598-023-38317-0)
Supplement: Supplementary file 1 — Supplementary Information. [file 41598_2023_38317_MOESM1_ESM.pdf]

Supplementary Information for

Extreme precipitation patterns in the Asia-Pacific region and its correlation with El Niño-Southern Oscillation (ENSO)

Dong An[1], Jakob Eggeling[2], Linus Zhang[1], Hao He[3], Amir Sapkota[4], Yu-Chun Wang[5] and Chuansi Gao[2]

[1]Division of Water Resources Engineering, Faculty of Engineering (LTH), Lund University, Sweden

[2]Aerosol and Climate Laboratory, Division of Ergonomics and Aerosol Technology, Department of Design Sciences, Faculty of Engineering (LTH), Lund University, Sweden

[3]Deptartment of Atmospheric and Oceanic Science, University of Maryland, College Park, MD 20742, United States

[4]Departement of Epidemiology and Biostatistics, University of Maryland, School of Public Health, College Park, MD 20742, United States

[5]Department of Environmental Engineering, College of Engineering, Chung Yuan Christian University, 200 Chung-Pei Road, Zhongli 320, Taiwan

*Correspondence to Dong An (dong.an@tvrl.lth.se)

Table 1 Number of locations with statistically significant / nonsignificant trends in extreme precipitation indices at annual scale over study area.

Extreme precipitation indices	95% (90%) * Significant increasing	Nonsignificant increasing ($p > 0.05$)	95% (90%) Significant decreasing	Nonsignificant decreasing ($p > 0.05$)
Rx1day	6 (8)	148	45 (72)	266
Rx5day	5 (7)	121	52 (74)	287
R95p	2 (6)	125	75 (119)	248
R99p	9 (16)	78	47 (85)	126
PRCPTOT	3 (7)	103	133 (167)	226
SDII	4 (7)	141	80 (105)	240
R10mm	5 (10)	84	117 (161)	198
R20mm	6 (11)	71	87 (114)	168
CDD	29 (53)	242	9 (14)	101
CWD	6 (9)	90	50 (81)	180
WetDays	0 (1)	90	125 (164)	214

* 95% significant level corresponds to $p \leq 0.05$, 90% significant level corresponds to $p \leq 0.10$. Same in Table 2.

Table 2 Number of locations with statistically significant/nonsignificant trends in seasonal maximum 1-day precipitation (*Rx1day*) and seasonal maximum 5-day precipitation (*Rx5day*) over study area.

	95% (90%) Significant increasing	Nonsignificant increasing ($p > 0.05$)	95% (90%) Significant decreasing	Nonsignificant decreasing ($p > 0.05$)
Rx1day_MAM	4 (17)	166	26 (53)	269
Rx1day_JJA	11 (17)	156	41 (66)	257
Rx1day_SON	9 (19)	217	21 (29)	218
Rx1day_DJF	2 (4)	172	22 (52)	269
Rx5day_MAM	5 (10)	149	48 (66)	263
Rx5day_JJA	14 (18)	129	42 (68)	280
Rx5day_SON	7 (13)	186	16 (36)	256
Rx5day_DJF	1 (4)	133	43 (75)	288

Table 3 Warm and cold phases for each season defined by Niño 3.4 SST index (1990–2019)

	MAM	JJA	SON	DJF
warm year	1992, 1993, 1998, 2015, 2016, 2019	1991, 1997, 2002, 2009, 2015	1991, 1994, 1997, 2002, 2004, 2006, 2009, 2014, 2015, 2018	1991, 1994, 1997, 2002, 2004, 2006, 2009, 2014, 2015, 2018, 2019
cold year	1999, 2000, 2008, 2011	1998, 1999, 2000, 2010	1995, 1998, 1999, 2000, 2007, 2010, 2011, 2016, 2017	1995, 1998, 1999, 2000, 2005, 2007, 2008, 2010, 2011, 2017

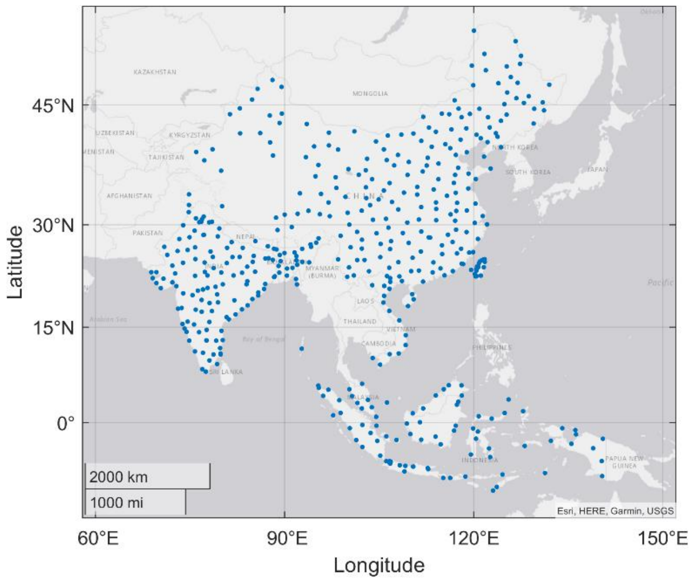

Figure 1. Blue dots indicate 465 study locations over the study area in eight countries and regions (9 in Bangladesh, 206 in China, 130 in India, 69 in Indonesia, 14 in Malaysia, 2 in Nepal, 15 in Vietnam, 20 in Taiwan region)

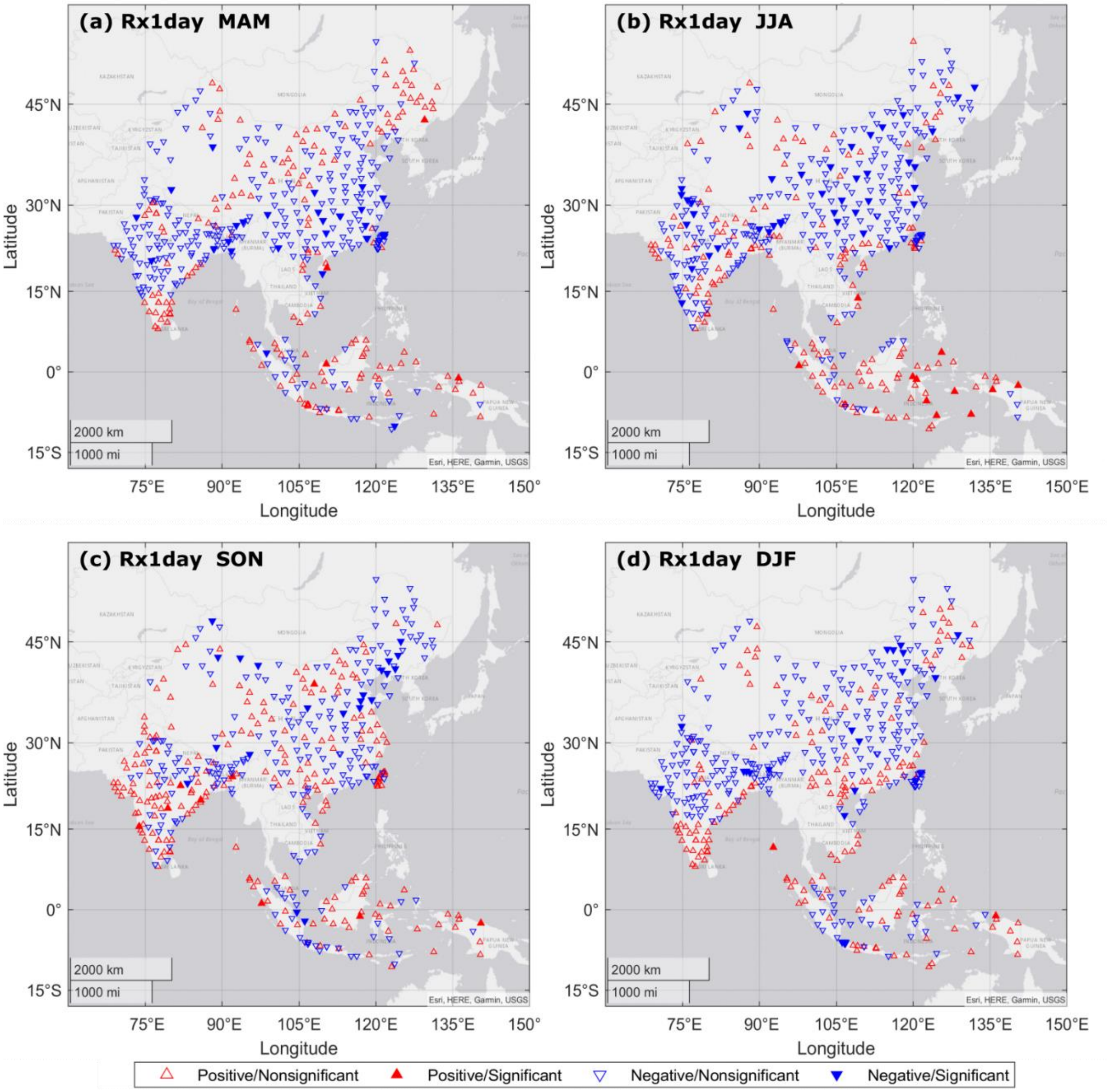

Figure 2. Spatial distribution of seasonal maximum 1-day precipitation (*Rx1day*) trends of (a) MAM, (b) JJA, (c) SON, and (d) DJF based on the iterative Mann-Kendall trend test. Upward (downward) triangles indicate positive (negative) trends, solid triangles indicate the trends at 95% significance level. Locations with no trends are not shown.

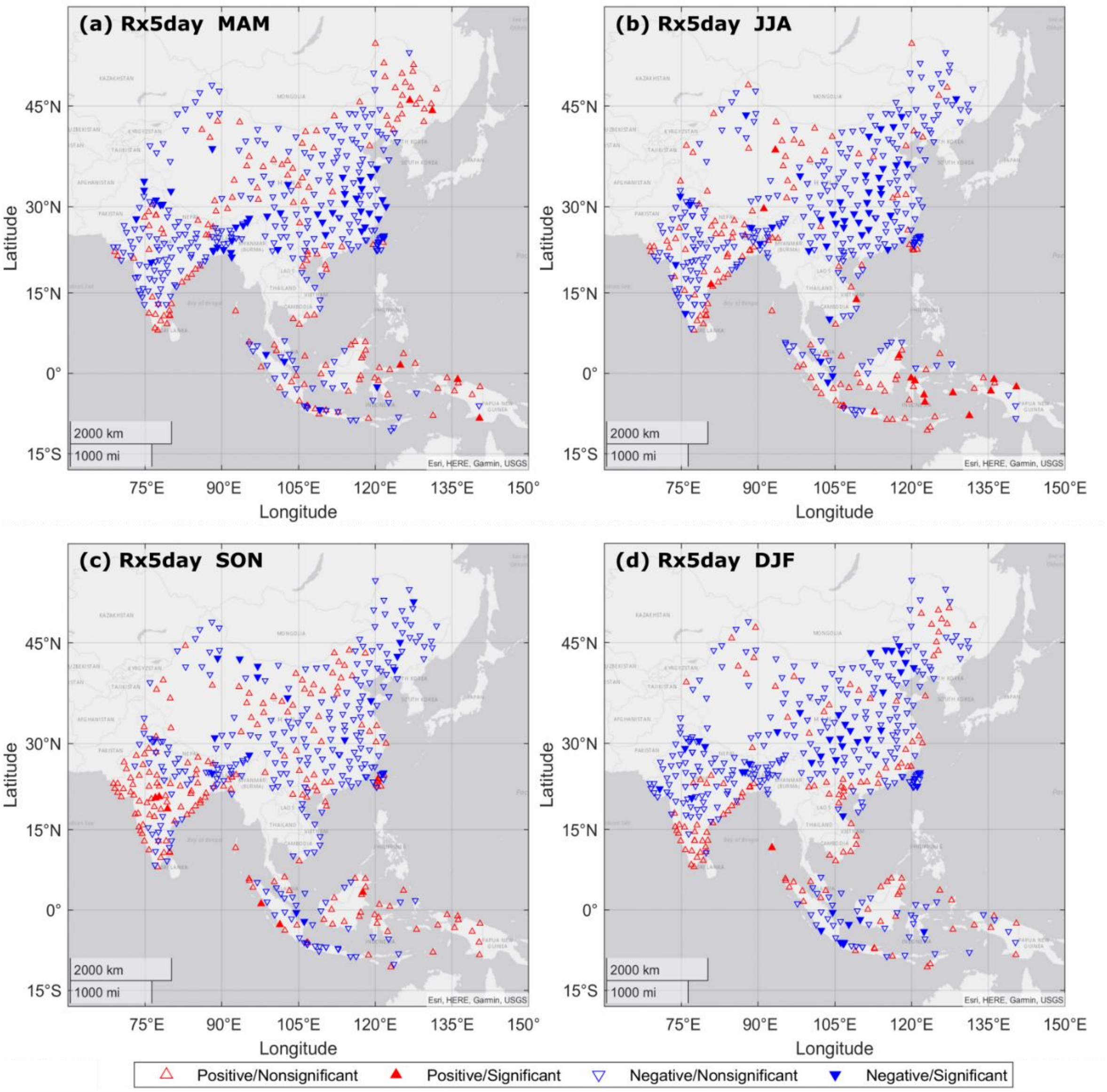

Figure 3. Spatial distribution of seasonal maximum 5-day precipitation (*Rx5day*) trends of (a) MAM, (b) JJA, (c) SON, and (d) DJF based on the iterative Mann-Kendall trend test. . Upward (downward) triangles indicate positive (negative) trends, solid triangles indicate the trends at 95% significance level. Locations with no trends are not shown.

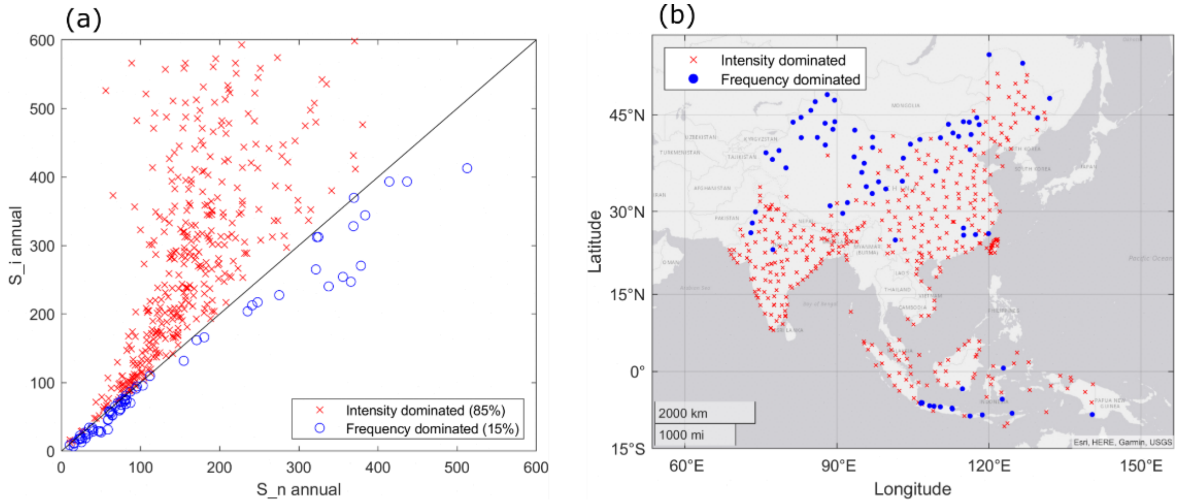

Figure 4. The (a) scatter plot of annual S_n and S_i and (b) spatial distribution of locations with dominances of intensity and frequency. The 45° line represents the condition S_n = S_i. Red cross indicates the dominance of precipitation intensity (S_i > S_n), blue dots indicates the dominance of precipitation frequency (S_n > S_i).
